# Comparison of serum 25-hydroxyvitamin D levels between patients with multiple chemical sensitivity and healthy controls: A case–control study

**DOI:** 10.1038/s41598-026-44643-w

**Published:** 2026-03-17

**Authors:** Kentaro Watai, Sae Ochi, Tomokazu Matsuura, Kenichi Azuma

**Affiliations:** 1 Department of Preventive Medicine and Behavioral Sciences, Kindai University Faculty of Medicine, 1-14-1 Mihara-dai, Minami-ku, Sakai, 590-0197 Osaka Japan; 2 Clinical Research Center for Allergy and Rheumatology, NHO Sagamihara National Hospital, Kanagawa, Japan; 3 Center for Immunology and Allergy, Shonan Kamakura General Hospital, Kanagawa, Japan; 4 Department of Laboratory Medicine, The Jikei University School of Medicine, Tokyo, Japan

**Keywords:** Multiple chemical sensitivity, Vitamin D, 25-hydroxyvitamin D, Biomarkers, Diseases, Medical research, Neurology, Neuroscience

## Abstract

Vitamin D deficiency has been associated with a range of neurological and allergic conditions. Whether such an association exists in multiple chemical sensitivity (MCS) has not been clarified. This study aimed to compare serum 25-hydroxyvitamin D (25[OH]D) concentrations between patients with MCS and healthy controls. We conducted a case–control study including 80 patients with physician-diagnosed MCS and 5,518 controls. Serum 25(OH)D concentrations were compared using a general linear model with bias-corrected and accelerated bootstrap resampling (1,000 iterations), adjusting for age, sex, season of blood collection, smoking status, body mass index, alcohol intake, and physical activity. Vitamin D deficiency (< 20 ng/mL) was highly prevalent in both groups (78.8% in MCS vs. 75.3% in controls). Median serum 25(OH)D concentrations did not differ significantly between groups (14.6 vs. 15.6 ng/mL, *p* = 0.622). Adjusted analyses confirmed no statistically significant difference (adjusted difference = 1.07 ng/mL, 95% CI: −0.18 to 2.46, *p* = 0.119). Despite the high prevalence of vitamin D deficiency, patients with MCS did not differ significantly from controls in serum 25(OH)D concentrations. Although serum 25(OH)D concentrations did not differ significantly between groups, the findings do not exclude a mechanistic role of vitamin D. Local dysregulation of vitamin D receptor signaling or tissue-specific activation may contribute to neuroimmune sensitization in MCS, highlighting the need for system-level investigations.

## Introduction

Vitamin D deficiency is associated with bone-related diseases as well as neurological dysregulation such as multiple sclerosis, mood disorders, and schizophrenia^1–6^. Furthermore, vitamin D deficiency is associated with allergic diseases such as asthma, atopic dermatitis, and allergic rhinitis^7–10^. This evidence suggests the possibility that vitamin D plays a role in neurological and allergic diseases. Multiple chemical sensitivity (MCS) shares the same aspects as neurological and allergic diseases. In terms of hypersensitivity, MCS is a disorder characterized by reactions to trace amounts of chemicals or environmental agents that healthy people do not react to, causing multisystem symptoms such as headache, dizziness, cough, and abdominal pain^11,12^. Additionally, MCS frequently co-occurs with allergic diseases such as allergic rhinitis^13,14^. From a neurological standpoint, MCS is a type of central sensitization syndrome in which brain or nerve hypersensitivity is thought to be involved^15,16^.

Recent neuroimmunological and clinical evidence suggests that MCS belongs to the spectrum of central sensitivity syndromes, characterized by altered neuroimmune signaling and persistent glial activation leading to heightened sensory responsiveness^15,17^. The concept of neuroimmune reflex pathways further supports this mechanistic link between peripheral inflammation and central sensitization^18^. Vitamin D, acting via vitamin D receptor (VDR) expressed in microglia and neurons may modulate this neuroimmune crosstalk^19,20^. The VDR activation suppresses nuclear factor kappa B (NF-κB)-driven pro-inflammatory gene expression^21^ and promotes tight-junction integrity of the blood–brain barrier (BBB). Thus, VDR signaling represents a molecular bridge linking vitamin D deficiency to neuroinflammation and toxicant susceptibility in MCS.

Vitamin D signaling, mediated through the VDR, regulates both innate and adaptive immune responses. VDR and the activating enzyme 1α-hydroxylase are expressed in neurons, astrocytes, microglia, and immune cells, indicating that vitamin D may act locally within the central nervous system to influence inflammatory activity^19,20^. In immune cells, active 1,25-dihydroxyvitamin D_3_ (1,25[OH]₂D₃) modulates cytokine production by down-regulating IL-1β and TNF-α and promoting IL-10–mediated anti-inflammatory responses^22,23^. Experimental studies also show that vitamin D reduces Toll-like receptors (TLR2 and TLR4) expression on monocytes and macrophages, attenuating TLR-triggered cytokine release^24,25^. We therefore hypothesize that insufficient vitamin D activity due to systemic deficiency may facilitate neuroinflammatory sensitization underlying MCS.

Considering the immunological and neurological aspects of vitamin D and MCS, it is important to clarify whether vitamin D is involved in the development of MCS. Although the association between vitamin D and MCS has been discussed from a neurological perspective^17^, a direct comparison of serum 25-hydroxyvitamin D (25[OH]D) levels between healthy individuals and individuals with MCS has not been reported. To this end, we aimed to compare the serum 25(OH)D status of MCS patients compared with that of healthy controls.

## Materials and methods

### Study design

**Fig. 1:**
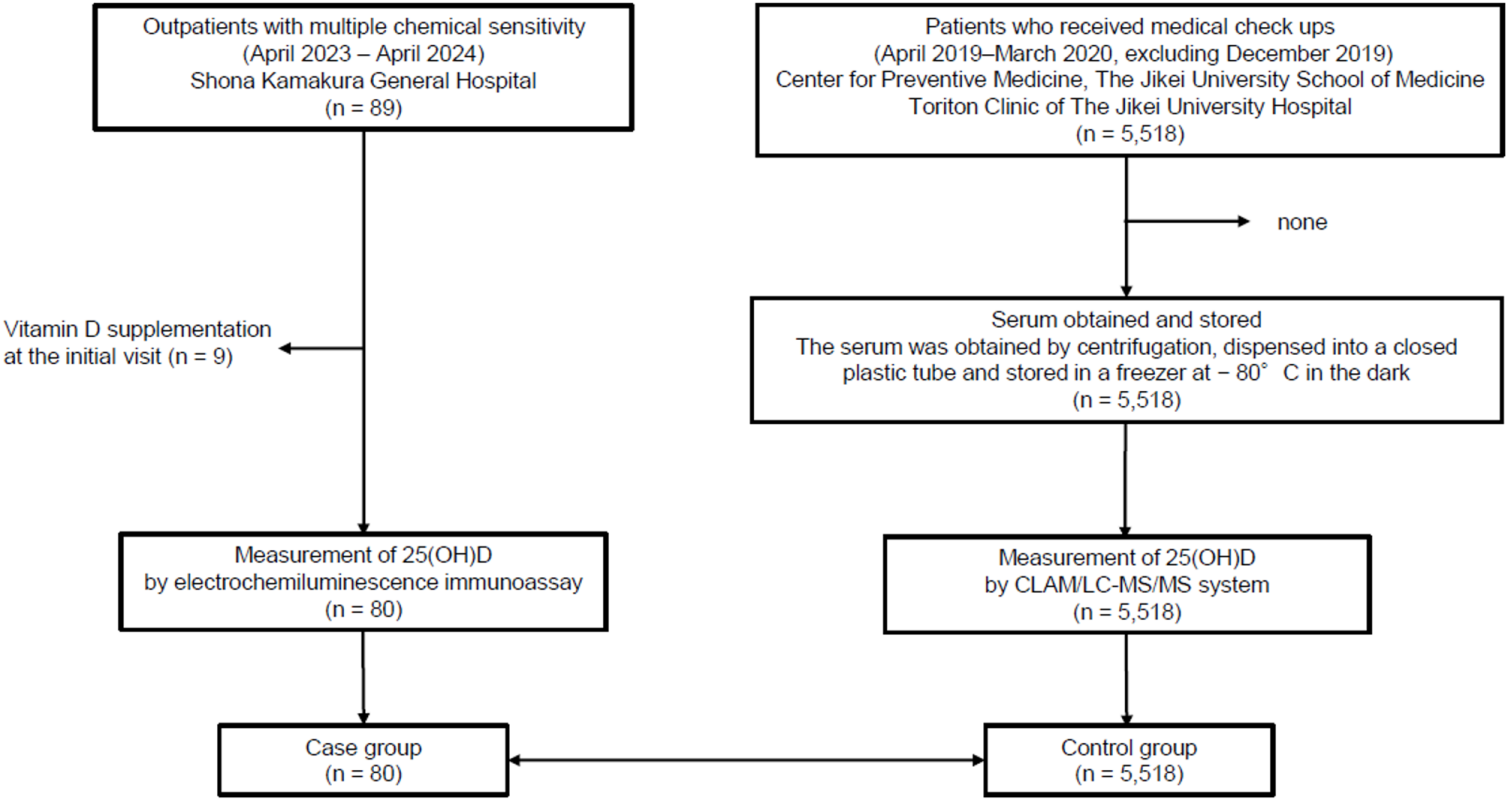
Participant flow diagram. CLAM: clinical laboratory automation module. LC–MS/MS: liquid chromatography–tandem mass spectrometry.

In this case–control study, we included consecutive patients diagnosed with MCS at the Immunology and Allergy Center of Shonan Kamakura General Hospital between April 2023 and April 2024 as the MCS group (purposive approach). For the control group, we included all consecutive individuals who underwent routine medical examinations at the Centre for Preventive Medicine, Jikei University School of Medicine, and the Toriton Clinic of Jikei University Hospital in Tokyo between April 2019 and March 2020 (excluding December 2019), and whose 25(OH)D levels were determined in a previous study (convenience approach)^26^. The case group (*n* = 80) and the control group (*n* = 5,518) were recruited from geographically adjacent areas (Tokyo and Kanagawa) located at similar latitudes to minimize differences in environmental UV exposure (Fig. 1).

All eligible patients diagnosed with MCS during the study period were consecutively included. In a post hoc power analysis based on the observed standard deviations (MCS group, SD = 6.0 ng/mL; control group, SD = 6.2 ng/mL), the available sample size (MCS, *n* = 80; controls, *n* = 5,518) provided 80% power to detect a between-group difference of ≥ 1.9 ng/mL in serum 25(OH)D levels at α = 0.05.

### Method for measuring serum 25 (OH) D

Serum 25(OH)D levels in the case groups were measured using an electrochemiluminescence immunoassay (ECLIA). For the control group, 25(OH)D levels were determined using a fully automated liquid chromatography–tandem mass spectrometry (LC–MS/MS) platform consisting of the Clinical Laboratory Automation Module (CLAM)−2030 sample preparation module and the liquid chromatography–mass spectrometry-8050 triple quadrupole mass spectrometer (both from Shimadzu Corporation). The correlation between ECLIA and LC–MS/MS methods has been verified in a previous study^26^(*r* = 0.949), and the equation used to determine this correlation is as follows: (y [ECLIA] = 1.000×[LC–MS/MS] + 0.850). As such, serum 25(OH)D levels of the two groups were compared based on their ECLIA values.

### Classification of 25(OH)D levels

Levels of 25(OH)D were classified as follows: sufficient (≥ 30 ng/mL), insufficient (20–29.9 ng/mL), and deficient (< 20 ng/mL)^27,28^.

### Diagnosis of multiple chemical sensitivity

The Quick Environmental Exposure and Sensitivity Inventory (QEESI) is a commonly utilized questionnaire with demonstrated high diagnostic accuracy for identifying MCS, exhibiting a sensitivity of 92% and specificity of 95%^29–32^. This tool has been applied in MCS research across several countries, including the United States, Japan, and Germany. A validated Japanese version of the QEESI is also available^30^. The instrument comprises five distinct domains: (1) chemical exposures, (2) other exposures, (3) symptoms, (4) masking index, and (5) impact of sensitivity. All sections, except the masking index, are scored on a 0–100 scale. Additionally, risk stratification criteria are applied to the chemical exposure (1), symptoms (3), and masking index (4) sections. Individuals scoring 40 points or more in both the chemical exposure and symptoms domains are categorized as “highly suggestive” of MCS, a criterion employed for participant selection in this study. Further methodological details are outlined below.

The QEESI is a self–reported assessment tool that captures individuals’ reactions to various environmental exposures, such as chemicals, food items, skin contactants, alcohol, and caffeine. Each of the first three scales — Chemical Exposures, Other Exposures, and Symptoms — comprises 10 items. Respondents rate their responses on a scale from 0 (no reaction) to 10 (severe reaction), based on symptom severity. The development and psychometric validation of these scales, including their sensitivity and specificity, have been described in earlier studies.

### Chemical exposures scale (1)

This domain evaluates participants’ sensitivity to ten diverse airborne substances. Participants assign a severity score from 0 to 10 for each item, which includes exposures such as exhaust from diesel or gasoline engines, tobacco smoke, pesticides, gasoline fumes, paints and thinners, scented products, cleaning agents, fresh asphalt or tar, nail products, hair spray, and new furniture.

### Symptoms scale (3)

This domain assesses a broad range of symptom categories, including head-related, musculoskeletal, respiratory and mucosal, cardiovascular, neuromuscular, gastrointestinal, cognitive, emotional, dermatologic, and genitourinary symptoms. Participants rate each symptom based on severity, where 0 indicates no symptoms, 5 indicates moderate symptoms, and 10 indicates severe or disabling symptoms. The total score for this scale ranges from 0 to 100, calculated by summing the scores of all 10 items.

To enhance the accuracy of case identification, both the QEESI criteria and physician-confirmed diagnoses were employed in determining MCS status among participants.

### Statistical analysis

Because serum 25(OH)D levels are influenced by demographic and lifestyle factors such as age, sex, season of blood collection, smoking status, body mass index, alcohol intake, and physical activity^26,33–35^, these variables were included as covariates in the general linear model with bias-corrected and accelerated bootstrap resampling. The distribution of serum 25(OH)D levels exhibited a slight leftward shift of the peak, resembling a log-normal distribution with noticeable deviation in skewness from a normal distribution. The normality of serum 25(OH)D distribution was tested using the Shapiro–Wilk test, which indicated deviation from normality (*p* < 0.001), supporting the use of nonparametric bootstrap estimation. Therefore, bias-corrected and accelerated (BCa) bootstrap resampling was employed, given its robustness in handling skewed data with SPSS software (version 30; IBM Corp, Armonk, NY, USA). Moreover, to ensure robustness, we applied BCa bootstrap resampling (1,000 iterations) to all general linear model estimates. This approach provides reliable confidence intervals even under violations of normality or unequal group variances, which may occur with small case samples. Potential confounders (age, sex, BMI, smoking, alcohol, physical activity, and season) were included as covariates. Multicollinearity was assessed using variance inflation factors < 5.0 for all variables, indicating no collinearity concerns.

The primary outcome of this study was the difference in serum 25(OH)D levels between patients with MCS and healthy controls. The secondary outcomes included the prevalence of vitamin D deficiency and the associations of demographic and behavioral factors with 25(OH)D concentrations. A p-value < 0.05 was considered statistically significant.

### Ethics approval

Shonan Kamakura General Hospital, the affiliation of the first author, is a member institution of the Tokusyukai Medical Group. This study was reviewed and approved by the Central Ethics Committee of the Tokusyukai Medical Group (approval No. TGE02540-024), and permission to conduct the study at Shonan Kamakura General Hospital was granted by the hospital director. All methods were performed in accordance with the relevant guidelines and regulations, including the Declaration of Helsinki and the Ethical Guidelines for Medical and Health Research Involving Human Subjects in Japan. For the case group, existing data obtained during routine clinical care were used, and consent was managed through an opt-out procedure in accordance with the applicable ethical guidelines. For the control group, written informed consent for the secondary use of data was obtained from all participants at the time of their health check-up.

## Results

Of 89 individuals included in the MCS cohort during the specified period, 9 individuals were excluded because of supplemental administration of vitamin D during the initial visit (Fig. 1). The control group included data from 5,518 individuals; however, their vitamin D supplement administration was unknown. In European countries and the United States, people actively consume vitamin D through supplements, and 25(OH)D₂ is often added to foods; therefore, the 25(OH)D₂ concentrations detected in populations in these regions are higher than those observed in the Japanese study^26^. In the data of controls, the 25(OH)D₂ levels in most participants were below the detection limit, suggesting that vitamin D supplements are not commonly used in this control group^26^. Therefore, in total, 80 cases and 5,518 controls were included in the analysis.

**Fig. 2:**
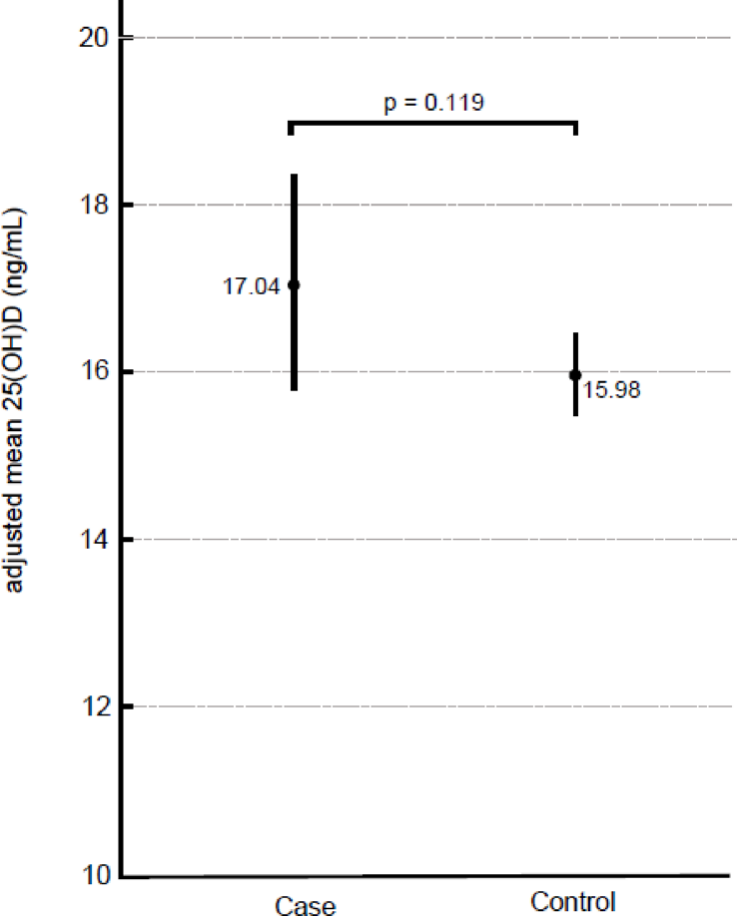
Adjusted means of serum 25(OH)D (ng/mL) by group with 95% confidence intervals (BCa bootstrap, 1,000 resamples). Bootstrap p-value for adjusted difference: 0.119. BCa, bias-corrected and accelerated bootstrap confidence interval.

Baseline demographic and lifestyle characteristics are summarized in Table 1. The MCS group consisted predominantly of females (77 of 80 participants, 96.3%), whereas males constituted a larger proportion of the control group (Table 1). We found that 78.8% of the individuals in the MCS group had vitamin D deficiency, and no significant difference was observed when compared with the rate in the control group (75.3%; Table 2). The serum 25(OH)D levels of the MCS group (measured by ECLIA) were not significantly different from that of the control group after conversion of LC–MS/MS values to ECLIA values^26^(median [interquartile range]: 14.6 [11.8–19.4] vs. 15.6 [12.0–20.0] ng/mL, *p* = 0.622]; Table 2). The adjusted analysis using a general linear model with bootstrap resampling (1,000 samples) showed no statistically significant difference in serum 25(OH)D₃ levels between the case and control groups after adjusting for sex, measurement season, smoking status, age, BMI, alcohol index, and physical activity (Difference = 1.07, 95% CI [BCa]: −0.18 to 2.46, *p* = 0.119; Table 3). Among the covariates, sex, age, time of blood sampling, and alcohol index were significant predictors of serum 25(OH)D levels, while BMI and physical activity were not. Smoking showed a borderline effect (*p* = 0.069; Table 3). Adjusted means were 17.04 ng/mL (95% CI [BCa]: 15.81–18.38) for the case group and 15.98 ng/mL (95% CI [BCa]: 15.48–16.45) for the control group, with an adjusted difference of 1.07 ng/mL (95% CI [BCa]: −0.18 to 2.46, *p* = 0.119; Table 4; Fig. 2).

**Table 1:** Demographics and serum 25(OH)D levels.

	Case group, *n* = 80	Control group, *n* = 5,518	*P* value
Sex, female/male	77/3	2,118/3,400	< 0.001
Age (years), median (IQR)	53 (43–65)	50 (39–59)	0.005
Time of blood sampling			
Winter (January to March), n (%)	16 (20.0)	600 (10.9)	< 0.001
Spring (April to June), n (%)	16 (20.0)	2,800 (50.7)	
Summer (July to September), n (%)	14 (17.5)	2,088 (37.8)	
Fall (October to December), n (%)	34 (42.5)	30 (0.5)	
Smoking [Current/(past + never)]	0/80	597/4,921	0.002
BMI (kg/m²)	22.6 (19.8–25.1)	22.4 (20.3–24.7)	0.874
Alcohol index (g/day)	0 (0–0)	0 (0–137)	< 0.001
Physical activity (min/day)	0 (0–0)	30 (0–60)	< 0.001

IQR, interquartile range; 25(OH)D, 25-hydroxyvitamin D.

† The values of the control group are adjusted values calculated using the following formula. The correlation between ECLIA and LC–MS/MS methods has been verified in the previous study^26^, *r* = 0.949 (*p* < 0.01), and the equation describing this correlation is y (ECLIA) = 1.000 x (LC–MS/MS) + 0.850.

Data are presented as median (IQR) or number (%) unless otherwise indicated. Between-group comparisons were performed using the Mann–Whitney U test or chi-square test as appropriate. A p-value < 0.05 was considered statistically significant.

**Table 2:** Serum 25(OH)D status in patients with MCS and healthy controls.

	Case group, *n* = 80	Control group, *n* = 5,518	*P* value
Levels of 25(OH)D			
25(OH)D, median (IQR), (ng/mL)	14.6 (11.8–19.4)	15.6 (12.0–20.0)^†^	0.622
Sufficient as ≥ 30 ng/mL, n (%)	2 (2.5)	145 (2.6)	0.765
Insufficiency as 20–29.9 ng/mL, n (%)	15 (18.8)	1,220 (22.1)	
Deficiency as < 20 ng/mL, n (%)	63 (78.8)	4,153 (75.3)	

IQR, interquartile range; 25(OH)D, 25-hydroxyvitamin D.

† The values of the control group are adjusted values calculated using the following formula. The correlation between ECLIA and LC–MS/MS methods has been verified in the previous study^26^, *r* = 0.949 (*p* < 0.01), and the equation describing this correlation is y (ECLIA) = 1.000 x (LC–MS/MS) + 0.850.

**Table 3:** General linear model with bootstrap.

Variables	B (Estimate)	95% CI (BCa)	Bootstrap *p*-value
Group (Ref. control) Case	1.07	−0.18 to 2.46	0.119
Sex (Ref. female) Male	2.11	1.77 to 2.43	0.001
Age (year)	0.077	0.064 to 0.088	0.001
Time of blood sampling [Ref. Winter (Jan to Mar)]			
Spring (Apr to Jun)	1.43	0.93 to 1.97	0.001
Summer (Jul to Sep)	1.15	0.62 to 1.69	0.001
Fall (Oct to Dec)	0.63	−1.11 to 2.21	0.448
Smoking (Ref. Current) Others (past + never)	−0.54	−1.14 to 0.04	0.069
BMI (kg/m²)	−0.033	−0.080 to 0.012	0.160
Alcohol index	0.001	0.000 to 0.002	0.014
Physical activity (min/day)	−0.001	−0.003 to 0.002	0.679

Dependent variable: Serum 25(OH)D, ng/mL.

Current smoking was used as the reference category; past and never smokers were grouped as “Others.”.

BCa, bias-corrected and accelerated bootstrap confidence interval (1,000 resamples); 25(OH)D, 25-hydroxyvitamin D.

A p-value < 0.05 was considered statistically significant. Bootstrap-based general linear models (1,000 iterations) were used for adjusted analyses.

**Table 4:** Adjusted means of serum 25(OH)D by group.

Group	Adjusted Mean (ng/mL)	95% CI (BCa)	*p*-value^†^
Case	17.04	15.81 to 18.38	
Control	15.98	15.48 to 16.45	
Difference (Case – Control)	1.07	−0.18 to 2.46	0.119

BCa, bias-corrected and accelerated bootstrap confidence interval (1,000 resamples); 25(OH)D, 25-hydroxyvitamin D,.

† Bootstrap p-value for adjusted difference.

A p-value < 0.05 was considered statistically significant.

Bootstrap-based general linear models (1,000 iterations) were used for adjusted analyses.

## Discussion

### Comparison of 25(OH)D levels between MCS patients and healthy controls

To our knowledge, this is the first study to compare 25(OH)D levels between patients with MCS and healthy controls. Although vitamin D deficiency was common in patients with MCS (78.8%), adjusted analyses showed no significant difference in 25(OH)D levels between patients and healthy controls. Our findings indicate that sex, age, and seasonal variation were observed to be associated with vitamin D status, whereas BMI and physical activity were not significant predictors. Interestingly, alcohol intake showed a modest positive association with serum 25(OH)D₃, while current smoking exhibited a borderline negative association. These results are consistent with previous reports highlighting the influence of seasonal sunlight exposure and age-related metabolic changes on vitamin D levels^34,36^. Age, sex, and comorbidities are well-established determinants of vitamin D metabolism. In our study, age and sex were significant predictors, consistent with prior reports indicating reduced dermal synthesis in aging and sex-specific behavioral differences in sun exposure and diet. Although comorbid conditions were not systematically collected, the exclusion of patients with active inflammatory diseases minimizes their potential influence^37–39^. The lack of a significant group effect suggests that the observed differences in vitamin D status may primarily reflect demographic and behavioral factors rather than disease status itself.

### Contradictory findings in previous studies

In a previous study, vitamin D levels in patients with multiple sclerosis show no significant difference compared to that of healthy controls, with both groups exhibiting low levels^40^. However, another study revealed significant differences, yielding contradictory results^41^. These discrepancies may be because 25(OH)D levels can vary depending on measurement conditions, such as age, sex, and seasonal variations in UV exposure^26^. Therefore, in this study, analyses were adjusted for sex, age, season of blood collection, smoking status, BMI, alcohol consumption, and physical activity. Moreover, the hospitals attended by the patient and control groups were in nearby areas, and the climate and the number of hours of sunlight were approximately the same.

### Potential role of vitamin D in inflammatory diseases

Research has suggested an inverse relationship between 25(OH)D levels and disease activity under various inflammatory conditions. Patients with rheumatoid arthritis have lower vitamin D levels than healthy controls, with a negative correlation between serum 25(OH)D levels and disease severity^42^. Similarly, in early inflammatory polyarthritis, higher 25(OH)D levels are associated with lower disease activity and C-reactive protein levels^43^. For ankylosing spondylitis, cross-sectional studies have indicated lower vitamin D levels in patients than in controls, with some evidence of an inverse correlation between 25(OH)D and disease activity^44^. Active patients with Behçet’s disease have significantly lower serum 25(OH)D levels than healthy controls and inactive patients^45^. These findings suggest a potential immunomodulatory role of vitamin D in inflammatory diseases, although longitudinal studies are needed to establish causality. While the relationship between MCS severity and serum 25(OH)D level is also interesting, we did not assess this relationship in this study. Notably, the assessment of MCS severity is subjective, as there are currently no established objective markers.

Vitamin D exerts broad immunomodulatory and neuroprotective effects. Mechanistically, 1,25(OH)₂D₃ inhibits NF-κB signaling, leading to downregulation of proinflammatory cytokines such as IL-1β, TNF-α, and IL-17^46–48^. Vitamin D promotes IL-10 production via VDR activation in immune and mast cells, thereby stabilizing mast cells and limiting histamine release relevant to MCS^49,50^.

Vitamin D further supports the differentiation and maintenance of regulatory T cells (Tregs) via FOXP3 gene activation^51^, contributing to restoration of immune tolerance. Simultaneously, the TLR–VDR–cytochrome P450 (CYP)27B1 axis provides a molecular link between innate toxin recognition and vitamin D–dependent resolution of inflammation^24^. Dysregulation of this pathway may result in persistent low-grade inflammation.

Within the central nervous system, VDR signaling preserves BBB integrity by maintaining tight junction proteins, including claudin-5, occludin, and zonula occludens-1^21^. VDR is also expressed in dopaminergic neurons and microglia, where it reduces glial activation and oxidative stress^19,20^. Hence, reduced vitamin D bioavailability could predispose individuals to chronic neuroinflammation and central sensitization.

Another important consideration is the interaction between environmental pollutants and vitamin D metabolism. Certain xenobiotics activate aryl hydrocarbon receptor pathway, which induces CYP24A1 expression and accelerates vitamin D degradation^52^. This mechanism may contribute to the paradox of low vitamin D bioactivity despite adequate sun exposure in individuals with high toxicant burdens. Environmental factors may also modulate vitamin D metabolism and signaling. Lipid-soluble toxicants could impair intestinal absorption by altering cholesterol-transport-mediated uptake pathways^53^. In addition, developmental or toxicant-induced changes in the cellular distribution of the VDR within neural tissue may attenuate local neuroprotective responses^54^. Together, these interactions suggest that disturbances in vitamin D absorption or receptor localization could amplify susceptibility to environmental neurotoxins.

Moreover, genetic polymorphisms of VDR may affect receptor expression and ligand affinity^55,56^. These differences could contribute to variability in treatment responses among patients with MCS.

CYP27B1 is expressed in various extrarenal tissues, including immune and epithelial cells, allowing local conversion of 25(OH)D to active 1,25(OH)₂D₃^57^. This paracrine/autocrine vitamin D activation plays a pivotal role in maintaining immune homeostasis at inflammatory sites. Reduced local CYP27B1 activity or impaired VDR signaling may enhance neuroimmune hypersensitivity. Thus, systemic 25(OH)D levels alone may not accurately reflect vitamin D activity at the tissue level.

Taken together, these mechanistic pathways suggest that vitamin D acts at multiple levels—immune, neural, and barrier functions—to modulate susceptibility to environmental triggers. Although correlative, these findings support future studies integrating serum biomarkers, VDR genotypes, and tissue-specific enzyme activity to clarify causal mechanisms in MCS.

Future studies should use a systems-level approach integrating neurobiology, immunology, and toxicology. This strategy may clarify whether targeted vitamin D modulation can reduce neuroinflammation and hypersensitivity in MCS.

### Uncertainty in the effectiveness of Vitamin D supplementation

To date, the results of randomized controlled trials have been inconsistent, and clear evidence of the effects of vitamin D is difficult to achieve^58,59^. With respect to the efficacy of vitamin D supplementation, the minimum effective serum 25(OH)D level is thought to vary with disease. This may be because the cut-off levels of serum 25(OH)D indicating deficiency or sufficiency may be disease-dependent, just as vitamin D metabolism is tissue-dependent^60^. Therefore, the relationship between vitamin D levels and disease activity remains unclear and may vary by disease. These insights provide important guidance for considering an appropriate approach for vitamin D supplementation therapy.

### Study limitations

This study has several limitations. First, despite a high reported correlation (*r* = 0.949) between ECLIA and LC–MS/MS, the use of different analytical platforms for cases and controls may have introduced minor systematic bias. Linear conversion assumes homoscedastic measurement error across the concentration range and does not ensure complete interchangeability between assays, even after correction. Moreover, immunoassays may show concentration-dependent systematic bias, particularly at lower concentrations. Furthermore, non-differential misclassification may have attenuated the between-group difference. To address this possibility, we conducted the following sensitivity analyses: (i) exclusion of extreme values (lowest and highest 5%), and (ii) comparison based on a three-category classification of serum 25(OH)D concentrations (deficient, insufficient, and sufficient) using ordinal logistic regression. The results of these sensitivity analyses were consistent with the primary analysis, and no statistically significant between-group differences were observed (data not shown). Future studies should employ a unified measurement method to eliminate this potential source of error. Second, the vitamin D supplementation status of the control group was unknown, which may have introduced residual confounding. However, based on the reference dataset, 25(OH)D₂ levels were below the detection limit in most participants, suggesting that supplement use was uncommon in this population. Future studies should include direct assessment of supplement intake to improve comparability between groups. Third, in the present study, the number of participants differed substantially between the patient group (*n* = 80) and the control group (*n* = 5,518). Although the applied statistical methods are robust to heteroscedasticity and unequal group sizes, statistical power is primarily limited by the small case group. As an additional sensitivity analysis, we performed bootstrap-based estimation after restricting the control group to 400 participants (1:5 relative to the 80 cases), and we observed no statistically significant difference in serum 25(OH)D levels between the two groups. Fourth, the MCS group consisted predominantly of women, whereas the control group included a higher proportion of men. Although sex was adjusted for in the statistical models, the very small number of male MCS cases limited our ability to assess potential effect modification by sex. Therefore, the present findings should be interpreted as being primarily applicable to female patients with MCS, and overgeneralization to male patients should be avoided. Notably, this sex distribution reflects the well-documented female predominance of MCS reported in epidemiological studies^13,14^.

Fifth, we adjusted for major demographic and lifestyle factors. However, other determinants of serum 25(OH)D levels, such as diet, comorbidities, and genetic polymorphisms, were not assessed. Furthermore, the approximately four-year gap between data collection for the control and case groups may have introduced secular effects (e.g., changes in lifestyle, diet, health awareness, or healthcare utilization), the influence of which cannot be completely excluded. We conducted an additional sensitivity analysis by restricting the re-analysis to samples collected during the spring and summer seasons. The adjusted between-group difference remained non-significant and was consistent with the results of the primary analysis (data not shown). Sixth, inflammatory biomarkers and objective indicators of toxicant exposure were not assessed in this study. The absence of these covariates may have limited our ability to evaluate the mechanistic links between vitamin D status, systemic inflammation, and environmental chemical burden. Seventh, sun exposure, which is a major determinant of vitamin D status, was not directly measured in this study. Although sun exposure generally influences serum 25(OH)D levels^61,62^, this association has not been consistently observed in patients with multiple sclerosis^40^. Therefore, the potential influence of sun exposure on our results cannot be completely excluded. Eighth, referral bias may have influenced case recruitment because patients were drawn from a tertiary allergy center. Despite multivariable adjustment, residual confounding cannot be completely excluded.

Finally, the case–control design restricts causal inference, and the cross-sectional assessment precludes evaluation of temporal or disease-severity–related associations between vitamin D status and MCS.

## Conclusion

In this case–control study, no significant differences in serum 25(OH)D levels were observed between patients with MCS and healthy controls after adjusting for demographic and lifestyle factors. While vitamin D deficiency was prevalent in both groups, our findings suggest that reduced vitamin D levels are unlikely to represent a disease-specific marker of MCS. Although no significant differences in serum 25(OH)D were observed between MCS patients and controls, this does not exclude mechanistic involvement. Vitamin D may exert regulatory effects at the local neuroimmune level rather than through systemic concentrations. Integrating neurobiology, immunology, and toxicology perspectives will be critical to elucidate how vitamin D signaling intersects with chemical exposure to influence central sensitization and chemical sensitivity. Given the limited sample size, observational design, and potential referral bias, these findings should be interpreted with caution. Future multicenter collaborative studies with larger, well-balanced samples and standardized statistical approaches are needed to validate these results.

## Data Availability

Due to ethical considerations, the dataset is not publicly available. However, pseudonymized data may be provided by the corresponding author upon a justified request.

## Abbreviations

1,25-dihydroxyvitamin D_3_: (1,25[OH]₂D₃)
25(OH)D: 25-hydroxyvitamin D
BBB: Blood–brain barrier
CLAM: Clinical laboratory automation module
CYP: Cytochrome P450
ECLIA: Electrochemiluminescence immunoassay
LC–MS/MS: Liquid chromatography–tandem mass spectrometry
MCS: Multiple chemical sensitivity
NF-κB: Nuclear factor kappa B
VDR: Vitamin D receptor
